# Factors affecting follicular emergence at 10 days postpartum in lactating dairy cows

**DOI:** 10.3168/jdsc.2025-0744

**Published:** 2025-05-12

**Authors:** Hiromi Kusaka, Takeshi Yamazaki, Minoru Sakaguchi

**Affiliations:** 1 Laboratory of Theriogenology, School of Veterinary Medicine, Kitasato University, Towada, Aomori 034-8628, Japan; 2 Hokkaido Agricultural Research Center, National Agriculture and Food Research Organization (NARO), Sapporo, 062-0045, Japan

## Abstract

Several factors influence postpartum ovarian activity in dairy cows. Our previous research confirmed a significant seasonal effect on the occurrence of early ovulation until 26 d postpartum, yet the relationships with earlier follicular development were not examined. This retrospective study aimed to describe the association between follicular development at 10 d postpartum and the presence of early ovulation in 536 lactations. Furthermore, the factors affecting the emergence of ≥15 mm (LL-size) of follicles at 10 d postpartum were assessed, including calving season, parity of dams, BW loss, period of years, number of calves, and uterine abnormality. Approximately 12% of cows lacked ≥10.0 mm follicles at 10 d postpartum and had a lower frequency of early ovulation (32.3%). Early ovulation was observed more in cows with at least one LL-size but no 10.0 to 14.9 mm follicles (69.7%). The odds ratio of LL-size follicle presence in the cows with high uterine abnormalities was significantly lower than in the cows with low uterine abnormalities (odds ratio: 0.20, 54.0% vs. 83.5%). Fewer <10.0 mm follicles were detected in the former than the latter. The cows that calved in summer had a higher frequency of LL-size follicles than those that calved in winter and spring. The effects of BW loss and dam parity on the presence of LL-size follicles approached significance. In conclusion, calving season, BW loss, and dam parity influenced the likelihood of LL-size follicle emergence. In particular, an abnormal uterine status 10 d after calving inhibited early follicular development, which might affect early ovulation.

## Highlights

• We characterized the earlier follicular development before early ovulation.
• Cows with follicles ≥15 mm in size were more likely to ovulate until 26 days postpartum.
• Uterine abnormal cows had a more negative impact on the earlier follicular development.

## Graphical Abstract

**Figure:**
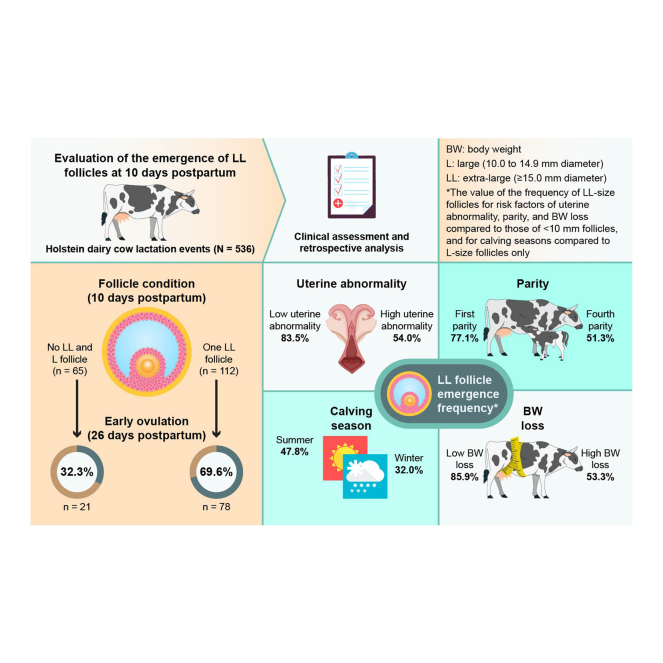
**Summary:** We investigated the association between follicular development at 10 days postpartum and the presence of early ovulation until 26 days postpartum. Furthermore, the factors affecting the emergence of ≥15 mm follicles at 10 days postpartum were assessed. Cows with no follicles ≥10 mm in diameter exhibit a lower frequency of early ovulation. In contrast, early ovulation was more frequently observed in cows with at least one ≥15 mm follicle. Cows with high uterine abnormalities had a significantly lower frequency of ≥15 mm follicles than those with low and medium uterine abnormalities. The cows that calved in summer had a higher frequency of ≥15.0 mm follicle emergence than those that calved in winter and spring. The body weight loss and the parity of dams were significantly associated with the frequency of ≥15.0 mm follicle emergence. Although various factors affect the emergence of ≥15 follicles, an abnormal uterine status may have a more direct and negative impact on postpartum follicular development than other factors.

The resumption of ovarian activity is essential for improving reproductive performance in subsequent breeding. Sequential ultrasonography examination revealed that 74% of 22 dairy cows ovulated after one follicular wave at ∼27 d postpartum, 5% ovulated after 3 waves, and 21% became cystic (Savio et al., 1990a). Similarly, the mean interval from parturition to first ovulation was 30.9 d postpartum, and 46% of the dairy cows ovulated at 18.1 d postpartum, on average, after one follicular wave developed (Sakaguchi et al., 2004). Most beef and dairy cows had first dominant follicles >8 mm in diameter, which is detected within 14 d postpartum. The follicle can ovulate within 4 wk postpartum in dairy cows but not in beef cows (Crowe et al., 2014). The fate of the first dominant follicle depends on LH pulse frequency. During the early postpartum period, failure to ovulate usually occurs due to inadequate LH pulse frequency and reduced ovarian follicle estradiol (Beam and Butler, 1999; Duffy et al., 2000; Cheong et al., 2016), which are associated with various factors in Holstein dairy cows.

Uterine bacterial infections usually occur during early postpartum in dairy cows (LeBlanc, 2008), and ∼90% of cows develop uterine bacterial infections within the first 2 wk following parturition (Sheldon et al., 2002). Failure to eliminate bacterial infection leads to uterine inflammation, which disrupts ovarian activity (Sheldon et al., 2002; Williams et al., 2007; LeBlanc, 2008). Sheldon et al. (2002) presented that when uterine bacterial growth scores were high 7 d postpartum, fewer first dominant follicles were selected in the ipsilateral than in the contralateral ovary to the side of the previous pregnancy. Williams et al. (2007) also showed that in animals with high numbers of uterine pathogens 7 d postpartum, the diameter of the first dominant follicle was smaller, plasma estradiol concentrations were lower, and they had a smaller corpus luteum that produced less progesterone. Severe inflammation of the postpartum uterus might impair follicular development and endocrine function.

Postpartum ovarian activity is also influenced by seasonality. Domestic cattle are not seasonal breeders but undergo several physiological changes in response to 2 major climatic variables: ambient temperature and photoperiod (Tucker, 1982). Hansen and Hauser (1983) focused on the photoperiod as a major seasonal climatic variable. Their study indicated that the exposure of primiparous cows to supplemental lighting altered postpartum ovarian activity. These seasonal differences are caused by variations in gonadotropin secretion (McNatty et al., 1984). Furthermore, a negative energy balance (**EB**) can inhibit postpartum ovarian activity, especially in dairy cows. After calving, high-producing dairy cows often experience a negative EB because they cannot fill the feed intake to meet the substantial extra demand for nutrients required for lactation (Esposito et al., 2014). The dominant follicles in cows under a negative energy status require more time and a larger size to establish blood estradiol concentrations capable of triggering ovulation by the LH surge (Beam, 1995). Aside from the disrupted hormone concentration, a recent study indicated that activated metabolites in negative EB cows may influence follicular development (Zhao et al., 2019). In addition, dam parity may influence the resumption of postpartum ovarian function; however, its effect is debated (Darwash et al., 1997; Tanaka et al., 2008; Balogh et al., 2009).

Our previous study using 542 lactation data evaluated the effects of calving season, dam parity, body condition, period of years, number of calves, and uterine abnormality on the occurrence of early ovulation until 26 d postpartum. In the previous study, the effect of calving season was the strongest, followed by body condition; thus, 71% of calving cows showed early ovulation during the summer, whereas 49%, 39%, and 47% presented this condition in autumn, winter, and spring, respectively (Sakaguchi et al., 2023). However, its effects on follicular development have not been examined before early ovulation. Furthermore, the risk factors for follicular development in lactating dairy cows have not been compared.

We hypothesized that different factors would be significantly associated with postpartum follicular development compared with early ovulation. Therefore, this study aimed to describe the association between follicular development at 10 d postpartum and the presence of early ovulation until 26 d postpartum. Furthermore, the factors affecting the emergence of ≥15 mm of follicles at 10 d postpartum were assessed, including calving season, dam parity, BW loss, period of years, number of calves, and uterine abnormality.

This retrospective study was conducted at the Hokkaido Agricultural Research Center, National Agriculture and Food Research Organization (NARO, Sapporo, Japan, latitude 41.28°N, with a subarctic climate), between October 1999 and June 2012 (annual average temperature; 8.3°C–9.8°C), as previously described (Sakaguchi et al., 2023). A total of 536 lactations (200 primiparous and 336 multiparous) of 249 Holstein dairy cows were examined. The average heifer age at the first service was 12 to 15 mo. All the cows were housed in the same freestall barn with a free-access soil paddock throughout the experimental period. The cows were fed a diet that met all maintenance, growth, and lactation requirements throughout the experimental period, following the Japanese feeding standards (Agriculture, Forestry and Fisheries Research Council Secretariat, 1999). During summer (May–September), the cows were pastured for 3 to 4 h per day, and the amount of food was reduced to meet the nutritional requirements necessary for this period. The cows were milked twice daily (0900 and 1900 h). The rolling herd average amount of milk per cow for sequential parities was 8,000 (first), 9,800 (second), 10,500 (third), and 10,700 kg (fourth and higher). Milk yield was standardized to a 305-d lactation length based on test-day records.

The postpartum uterine status was observed by transrectal palpation (**TP**) at 10 d postpartum (mean ± SD: 10.1 ± 0.9 d). We recorded the size of the uterus and the volume of vaginal discharge at that time. The status was graded on a scale of 0 to 2 as a TP score based on previous reports (0 = uterus returning to normal size with no discharge; 1 = enlarged uterus with a low-volume discharge, which was off-white or mucopurulent material; 2 = enlarged uterus with a high-volume discharge that was reddish, red-brown material; Noakes et al., 2019). After that, the uteri of all cows were monitored using a real-time linear array ultrasound scanner (SSD 620 with a 5-MHz probe or SSD-900 with a 7.5-MHz probe; Aloka, Tokyo, Japan). The amount and characteristics of intrauterine fluid were recorded. Intrauterine fluid was also categorized on a 0 to 2 scale as an ultrasound examination (**UT**) score based on a previous study (0 = no or anechogenic fluid; 1 = echogenic compact content; 2 = echogenic fluctuant content; López-Helguera et al., 2012).

The ovaries of all cows were also monitored by ultrasonography at 10 d postpartum. The number and size of the follicles were recorded. The total frequency of the emergence of ≥15.0 mm diameter follicles in 536 lactating periods was 31.7%. At 26 d postpartum (26.0 ± 0.7 d), we rechecked the ovaries, and the presence or absence of corpus luteum (**CL**; diameter >10 mm) was confirmed for each cow. In our previous study, the mean interval from calving to first ovulation was 30.9 d (Sakaguchi et al., 2004), with a median of 26 d in Holstein cows. Early ovulation was defined as the presence of the CL on d 26. Before these examinations, clinical signs such as fever or loss of appetite were not confirmed in the cows enrolled in this study. Only one veterinarian sampled these data throughout the experiment period.

All statistical analyses were performed using the JMP statistical software (JMP Pro Statistics and Graphics Guide ver. 18.0.2; SAS Institute Inc., Cary, NC). A *P* ≤ 0.05 was considered statistically significant, and *P* > 0.05 and *P* ≤ 0.10 were considered approaching significance. Continuous variables are presented as mean ± SD. As in the study of Beam and Butler (1997), the follicles at 10 d postpartum were categorized into the following 3 classes by size: medium (5.1–9.9 mm, **M**), large (10.0–14.9 mm, **L**), and extra large (≥15.0 mm, **LL**). Using a combination of the number of L- and LL-size follicles, follicular emergence patterns were characterized into 4 groups: neither L- nor LL-size follicles, at least one L- but no LL-size follicles, at least one LL- but no L-sized follicles, and >L-sized follicles. Differences in the frequency of early ovulation were evaluated among the 4 groups using the χ^2^ test. Multivariable logistic regression analysis was used to examine the effect of follicular emergence 10 d postpartum on the occurrence of early ovulation. These 4 combination groups were included as explanatory variables. Odds ratios (**OR**) and 95% CI were calculated.

A multivariable logistic regression model was used to evaluate factors influencing the presence of ≥15.0 mm follicles. The response variables were the presence of LL-size follicles (n = 171) and that of only L-size follicles (n = 300) in model 1, or they were the presence of LL-size follicles and that of only M-size follicles (n = 65) in model 2. In both models, the explanatory variables were selected based on previous studies. Calving season and parity were classified into 4 categories: calving season: spring (April–June), summer (July–September), autumn (October–December), and winter (January–March; Hansen and Hauser, 1983) and parity: 1, 2, 3, and ≥4 (Morales Piñeyrúa et al., 2018). Body weight loss, calculated as the difference from 1 wk before to 1 wk after calving, was used to examine the effect of postpartum energy status (Sakaguchi, 2009). The weight of the calf, placenta, and associated fluids were included in the BW of dams before calving but not after. The percentage of BW loss was grouped into 3 categories: low (5.6 ± 1.7%, 40.3 ± 13.4 kg), medium (9.6 ± 0.1%, 69.2 ± 11.6 kg), and high (14.1 ± 2.7%, 102.3 ± 24.1 kg). The number of calves delivered per cow was categorized into 2 grades: single and twin. The postpartum uterine abnormality score was determined using the combination of TP and UT scores: low (0–0, 1–0, and 0–1), intermediate (1–1, 2–0, and 0–2), high (2–1, 1–2, and 2–2). To consider genetic changes throughout the experimental period, the year was included in the analysis, which was grouped into 4 categories: period 1 (1999–2002), period 2 (2003–2005), period 3 (2006–2008), and period 4 (2009–2012). This model did not include the interaction between calving seasons, BW loss, and abnormal uterine scores because there was no significant effect. The largest follicle diameter and the number of <10.0 mm follicles were compared using Tukey's range test between the 3 grades of uterine abnormalities.

Table 1 presents the combination of the number of L- and LL-size follicles and their relationship with early ovulation by 26 d postpartum. Approximately 12% of cows did not have ≥10 mm diameter of follicles. The most common combination pattern for L- and LL-size follicles was 1–0, and the second was 0–1. The frequency of early ovulation differed significantly among the 4 groups (*P* < 0.01). Cows with no L-size follicles had a smaller frequency of early ovulation (32.3%), whereas early ovulation was observed more in the cows with at least one LL but no L follicles (69.7%) than other groups. According to multivariable logistic regression analysis, the highest OR for early ovulation was obtained in the group with at least one LL-sized follicle, compared with the group with no L-size follicles (OR: 4.83, *P* < 0.01). The FSH surge began immediately after parturition, and it peaked around 3 to 5 d postpartum and decreased until 8 d postpartum; thus, follicles >10 mm in diameter are typically detected around 10 d postpartum (Savio et al., 1990b; Beam and Butler, 1997). Our results indicated that the cows, having ≥15.0 mm first dominant follicles at 10 d postpartum, often had early ovulation at 26 d postpartum.

**Table 1:** Combination of the number of L^1^- and LL^2^-size follicles and the relationships of those with early ovulation^3^ by postpartum 26 d

Group	Number of follicles L^1^ and LL^2^	% (n) of the occurrence of early ovulation^3^	OR	*P*-value
Neither L nor LL class follicles	0–0	32.3 (21/65)	Referent	
At least one L but no LL follicles	1–0	45.9 (94)		
	2–0	48.1 (38)		
	3–0	71.4 (10)		
	4–0	50.0 (1)		
	Total	47.7 (143/300)	1.91	0.02
At least one LL but no L follicle	0–1	69.6 (78)		
	0–2	71.4 (5)		
	Total	69.7 (83/119)	4.83	<0.01
>L class follicles	1–1	59.2 (29)		
	2–1	50.0 (1)		
	1–2	0.0 (0)		
	Total	57.7 (30/52)	2.86	0.01

1 L-size, 10.0–14.9 mm follicle in diameter.

2 LL-size, ≥15.0 mm follicle in diameter.

3 Early ovulation was defined as the presence of a corpus luteum at 26 d postpartum.

An abnormal uterine status may strongly inhibit follicular development compared with other factors in the present study (Table 2). The OR of LL-size follicle presence in the cows with high uterine abnormalities was significantly lower than in the cows with low uterine abnormalities (OR: 0.20, *P* < 0.01, Table 3). Additionally, fewer <10.0 mm follicles were detected in cows with high uterine abnormalities than those with low and intermediate abnormality scores (2.7 vs. 3.4 and 3.5 follicles, *P* < 0.05). These adverse effects of uterine abnormalities on follicular development may be associated with postpartum bacterial contamination in the uterus, which causes slower follicular growth and decreased estradiol production (Sheldon et al., 2002; Williams et al., 2007). Bacterial endotoxins, such as LPS, can directly suppress follicular activity, irrespective of the developmental stage of the large follicles (Magata et al., 2014). Furthermore, follicular fluid LPS concentration may be causative for nonovulatory dairy cows (Cheong et al., 2017). Although the uterine bacterial load score was not examined in the present study, bacterial contamination might strongly inhibit follicular development during the early postpartum period.

**Table 2:** Results from multivariable logistic regression analysis for the presence of LL-size follicles against that of only L-size follicles (model 1) or against that of only M-size follicles (model 2)

Effect	df	Model 1		Model 2	
		LR^1^ χ^2^	*P*-value	LR^1^ χ^2^	*P*-value
Calving season^2^	3	10.5	0.02	3.11	0.37
Dam parity^3^	3	7.29	0.06	6.92	0.07
Period of years^4^	3	3.38	0.34	1.03	0.79
BW loss^5^	2	1.79	0.41	5.20	0.07
Number of calves	1	0.72	0.40	4.58	0.03
Uterine abnormality level^6^	2	13.1	<0.01	14.9	<0.01

1 LR = likelihood ratio.

2 Summer: July–September; autumn: October–December; winter: January–March; spring: April–June.

3 1, 2, 3, and ≥4.

4 Period 1: 1999–2002; period 2: 2003–2005; period 3: 2006–2008; period 4: 2009–2012.

5 Low: 5.6 ± 1.7%; medium: 9.6 ± 0.1%; high: 14.1 ± 2.7%.

6 Low (≤1), intermediate (2), and high (≥3) values were obtained from the sum of postpartum uterine evaluation scores using transrectal palpation and ultrasonography.

**Table 3:** Odds ratio for the presence of ≥15.0 mm against the absence of ≥10 mm follicles

Factor	Category	% (n)	Odds ratio	95% CI	*P*-value
Calving season^1^	Summer	73.3 (55/75)	Referent		
	Autumn	79.6 (39/49)	1.3849	0.5151–3.7238	0.519
	Winter	70.2 (40/57)	0.6556	0.2721–1.5818	0.348
	Spring	67.3 (37/55)	0.6369	0.2658–1.5262	0.312
Dam parity	1	77.1 (64/83)	Referent		
	2	80.0 (56/70)	1.1851	0.5110–2.7487	0.692
	3	70.5 (31/44)	0.7098	0.2751–1.8309	0.478
	≥4	51.3 (20/39)	0.3395	0.1323–0.8714	0.025
Period of years^2^	Period 1	73.6 (39/53)	Referent		
	Period 2	68.1 (49/72)	0.8788	0.3546–2.1778	0.780
	Period 3	74.6 (41/55)	1.3495	0.5008–3.6370	0.553
	Period 4	75.0 (42/56)	1.1975	0.4460–3.2156	0.721
BW loss^3^	Low	85.9 (55/64)	Referent		
	Medium	72.4 (92/127)	0.4662	0.1981–1.0974	0.081
	High	53.3 (24/45)	0.3241	0.1152–0.9115	0.033
Number of calves	Single	74.8 (169/226)	Referent		
	Twin	20.0 (2/10)	0.1603	0.0265–0.9683	0.046
Uterine abnormality level^4^	Low	83.5 (66/79)	Referent		
	Intermediate	75.5 (71/94)	0.5912	0.2587–1.3509	0.213
	High	54.0 (34/63)	0.2015	0.0844–0.4815	<0.0001

1 Summer: July–September; autumn: October–December; winter: January–March; and spring: April–June.

2 Period 1: 1999–2002; period 2: 2003–2005; period 3: 2006–2008; and period 4: 2009–2012.

3 Low: 5.6 ± 1.7%; medium: 9.6 ± 0.1%; high: 14.1 ± 2.7%.

4 Low (≤1), intermediate (2), and high (≥3) values were obtained from the sum of postpartum uterine evaluation scores using transrectal palpation and ultrasonography.

Domestication has turned cattle into annual breeders; however, seasonality in the resumption of ovarian activity may persist, as shown in our previous study. Compared with animals that calved in summer, the frequency of early ovulation was significantly lower in cows that calved in winter, spring, and autumn (Sakaguchi et al., 2023). Similarly, a significant impact of calving seasons was detected in the presence of LL-size follicles (Table 2). The cows that calved in summer had a higher frequency of LL-size follicle emergence than those that calved in winter and spring (47.8% vs. 32.0% and 29.6%, *P* < 0.01). Plasma concentrations of LH and prolactin differ by season, resulting in seasonal differences in ovarian activities, such as the pattern of follicular atresia and the diameter of healthy antral follicles (McNatty et al., 1984). Plasma prolactin concentration positively correlates with day length and ambient temperature from spring to summer and varies with the frequency of LH pulses (Tucker, 1982; McNatty et al., 1984). Our results also indicated that the calving season significantly influenced the presence of LL-size follicles against that of L-size but not M-size follicles (Table 2). Thus, we speculate that the seasonal changes in LH pulse frequency might influence the development of larger follicles that have acquired dominance.

Negative EB cows had a weaker follicular growth rate at 8 wk postpartum than positive EB cows (Song et al., 2021). Metabolic stress, which often causes negative EB, could be an important cause of reduced LH pulse frequency (Cheong et al., 2016). In addition, the increase in intrafollicular concentration of BHB, commonly elevated in negative EB cows, strongly affected follicular growth (Missio et al., 2022). In the present study, the effect of BW loss on the presence of LL-size follicles approached significance in model 2 (*P* = 0.07, Table 2). The OR of LL-size follicle presence in high BW loss cows was significantly lower than in low BW loss cows (OR: 0.32, *P* = 0.03, Table 3). The absence of >10.0 mm follicles might reflect a more severe energy status after parturition. However, this study could not accurately evaluate BW loss in dams because the weight of the conceptus was not fully considered. Therefore, BW loss, as a parameter of nutritional status, may require further refinement.

Postpartum follicular development is affected by parity (Tanaka et al., 2008; Zhang et al., 2010). In previous studies investigating postpartum follicular dynamics, primiparous—but not multiparous—cows exhibited repetitious follicular waves of nonovulatory follicles, which resulted in delayed first ovulation compared with multiparous cows (Tanaka et al., 2008; Zhang et al., 2010). These findings suggest that greater nutritional demands placed on younger cows, due to concurrent growth and lactation, may contribute to postpartum abnormal ovarian activity. In contrast, the present study observed that cows with parity ≥4 had significantly lower frequencies of LL-size follicles compared with first-parity cows (51.3% vs. 77.1%, *P* = 0.03; Table 3), a pattern not observed in cows of parity 2 or 3. Generally, the body growth of Holstein cows is completed by the third calving, marking full physiological maturity (Coffey et al., 2006). It is speculated that the lower prevalence of large follicles in cows with parity ≥4 may reflect changes in ovarian activity after reaching physiological maturity.

Although the calving season, dam parity, and BW loss influenced the chance of the emergence of ≥15.0 mm follicles, an abnormal uterine status may have a more direct and negative impact on follicular development than other factors. Less emergence of ≥15.0 mm follicles might cause the reduction of the occurrence of early ovulation. Postpartum uterine status may affect fertility through several mechanisms, including influencing the probability of ovulation, oocyte competence, and subsequent early embryo quality, development, and survival (LeBlanc, 2023). High uterine abnormalities, resulting in the absence of ≥15.0 mm follicles around postpartum 10 d, may be associated with subsequent poorer fertility. These results suggest that it might be important to manage the early postpartum uterus to improve reproductive performance.

The risk factors for the resumption of ovarian activity in dairy cows have attracted much interest; however, these factors are challenging to assess because of the presence of various confounders and interactions among the factors. Nutritional management could change with seasons and over the years, which is related to postpartum energy status. In addition, that energy status could affect the immune function of the postpartum uterus (Wathes et al., 2009). Although significant interaction among BW loss, calving season, and uterine abnormalities were not detected in this experimental herd, it might be detected in other herds. Additionally, in our study, cows that delivered twins had a lower frequency of LL-size follicles than those that delivered single calves (Table 3). Twin-calved cows may have experienced more severe weight loss and greater uterine damage after calving compared with single-calved cows. Thus, the impact of twin-calving may reflect the combined effects of multiple factors. Furthermore, because of genetic improvements, Holstein dairy cows produce vast amounts of milk, affecting the resumption of postpartum ovarian activity (Crowe et al., 2014). It was supposed that the period might affect the emergence of LL-size follicles, but this was not detected. Further studies with a larger sample size are required to clarify those interactions.

In conclusion, the presence of follicles with ≥15.0 mm in diameter at 10 d postpartum was significantly associated with early ovulation until 26 d postpartum. The calving season, dam parity, and BW loss influenced the chance of the emergence of ≥15.0 mm follicles. An abnormal uterine status at postpartum 10 d could have a stronger negative impact on the emergence of ≥15.0 mm follicles than other factors. These findings highlight the impact of factors affecting follicular development in lactating dairy cows during the early postpartum period. Determining the relationships of these factors can lead to improvements in the resumption of ovarian activity in subsequent breeding.
